# Diffuse large B-cell lymphoma in the liver accompanied by primary biliary cholangitis: A rare and difficult-to-diagnose tumor with portal venous thrombosis

**DOI:** 10.1016/j.ijscr.2021.105936

**Published:** 2021-04-30

**Authors:** Hikotaro Katsura, Tomohide Hori, Hidekazu Yamamoto, Hideki Harada, Michihiro Yamamoto, Masahiro Yamada, Takefumi Yazawa, Ben Sasaki, Masaki Tani, Asahi Sato, Yasuyuki Kamada, Ryotaro Tani, Ryuhei Aoyama, Yudai Sasaki, Masazumi Zaima

**Affiliations:** Department of Surgery, Shiga General Hospital, Moriyama, 5-4-30 Moriyama, Moriyama, Shiga 524-8524, Japan

**Keywords:** Diffuse large B-cell lymphoma, Liver, Malignant lymphoma, Primary biliary cholangitis, Thrombosis, Portal vein

## Abstract

**Introduction and importance:**

The most common liver malignancies are hepatocellular carcinoma, intrahepatic cholangiocarcinoma, and metastatic tumors. Hepatocellular carcinoma and intrahepatic cholangiocarcinoma may invade the portal vein (PV). An association between diffuse large B-cell lymphoma (DLBCL) and primary biliary cholangitis (PBC) remains unclear. We herein report a thought-provoking case of a difficult-to-diagnose liver tumor with PV thrombosis in a PBC patient.

**Presentation of case:**

A 66-year-old woman had PBC, systemic sclerosis, diabetes, and osteoporosis. A solitary liver tumor accompanied by macrovascular thrombosis in the PV was detected incidentally. Based on dynamic imaging findings, we considered the tumor to be intrahepatic cholangiocarcinoma, and right lobectomy with lymphadenectomy was performed. Unexpectedly, pathological assessment made a definitive diagnosis of DLBCL that did not invade the vessels and bile duct. In fluorine-18-fluorodeoxyglucose positron emission tomography, abnormal accumulations were clearly observed in the breast tissue and peritracheal, parasternal, mediastinal, and pericardial lymph nodes. The patient achieved complete remission after systemic chemotherapy, and there has been no recurrence 3 years after surgery.

**Clinical discussion:**

Primary lymphoma in the liver is rare, and we did not consider our patient's tumor as primary liver lymphoma. Our case actually showed no tumor thrombosis in the PV. Although autoimmune disorders may increase the risk of non-Hodgkin's lymphoma, an association between DLBCL and PBC is still unclear, and we must remember that DLBCL may develop rarely in a PBC patient.

**Conclusion:**

Our case report provides a timely reminder for clinicians and surgeons in the fields of hepatology and hematology.

## Highlights

• The most frequent liver neoplasms are HCC, ICC, and metastatic tumors.
• HCCs often cause portal thrombosis.
• Primary liver lymphoma is rare, and generally does not invade the vessels.
• An association between diffuse large B-cell lymphoma and PBC remains unclear.
• We describe a thought-provoking case of a difficult-to-diagnose liver tumor in PBC.

## 1 Introduction

Regarding liver malignancies, the most common tumors are two primary liver neoplasms (*i.e.*, hepatocellular carcinoma [HCC] and intrahepatic cholangiocarcinoma [ICC]) and metastatic tumors [1]. Diffuse large B-cell lymphoma (DLBCL) is the most common subtype of non-Hodgkin's lymphoma [2]. Autoimmune disorders may increase the risk of lymphoma [2,3], but an association between DLBCL and primary biliary cholangitis (PBC) is unclear [2]. We herein describe a thought-provoking case of a difficult-to-diagnose liver tumor with portal venous (PV) thrombosis in a PBC patient. This case was reported in accordance with the SCARE 2020 Guideline [4].

## 2 Presentation of case

The patient was a 66-year-old woman. She had a history of PBC, systemic sclerosis, type 2 diabetes, osteoporosis, and two autoimmune diseases. She received continuous oral medications, namely ursodeoxycholic acid, tapered prednisolone, and antidiabetic agents. Additionally, serum glycated hemoglobin was elevated (6.5%). She had no history of drinking alcohol, and hepatitis B and C viral infections were not observed. She had a family history of gallbladder cancer. Periodic ultrasonography of the liver detected a solitary tumor 3 cm in size located in the right posterior segment, accompanied by macrovascular thrombosis in the PV. She was referred to our hospital for detailed investigation and subsequent treatment.

**Fig. 1:**
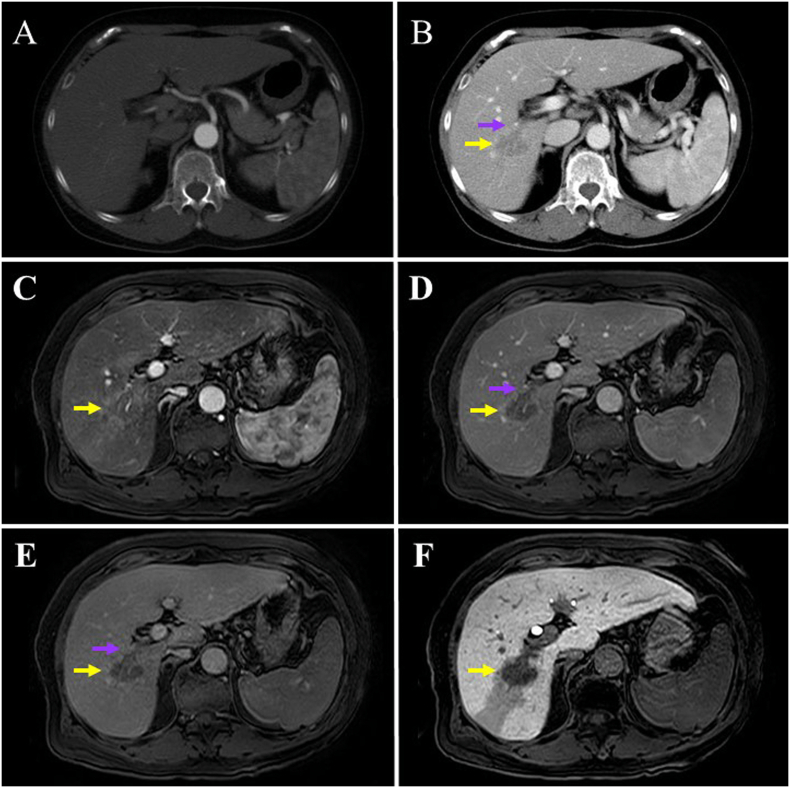
Dynamic computed tomography and magnetic resonance imaging findings. The images show a solitary tumor measuring 3 cm in size (yellow arrows) accompanied by portal vein (PV) thrombosis (suspicious tumor thrombosis) (purple arrows). In dynamic computed tomography, the tumor is enhanced during the arterial phase (A), and only subtly enhanced during the portal phase (B). In dynamic magnetic resonance imaging, the tumor is unenhanced during the arterial phase (C), and only subtly enhanced during the portal (D) and parenchymal (E) phases. The tumor is visible as a clear defect during the parenchymal phase in gadolinium-ethoxybenzyl-diethylenetriamine-pentaacetic acid-enhanced magnetic resonance images (F). (For interpretation of the references to colour in this figure legend, the reader is referred to the web version of this article.)

Serum concentrations of the tumor markers alpha-fetoprotein, protein induced by vitamin K absence or antagonist-II, carcinoembryonic antigen, and carbohydrate antigen (CA) 19-9 were within their respective normal ranges. Endoscopic examinations of the digestive tract were performed to rule out metastatic liver tumor, and no gastric and colorectal tumors were detected. Dynamic computed tomography and magnetic resonance imaging revealed that the solitary tumor showed no enhancement during the arterial phase, and only subtle enhancement during the portal and parenchymal phases (Fig. 1A–E). Hence, the typical appearance of early wash-in and wash-out of contrast agent was not observed. The tumor was recognized as a remarkable defect during the parenchymal phase in gadolinium-ethoxybenzyl-diethylenetriamine-pentaacetic acid-enhanced magnetic resonance imaging (Fig. 1F). No lymph node (LN) enlargement was observed in thoracic and abdominal imaging studies. We made a preoperative diagnosis of ICC (not HCC) accompanied by tumor thrombosis in the PV, and categorized the tumor as T2N0M0 stage II according to the tumor-node-metastasis classification [5].

In the indocyanine green clearance test, the elimination rate at 15 min and the *k* value were 10.2% and 0.154, respectively; the Child–Pugh score was 5 points. Moderate splenomegaly was observed; however, other findings of portal hypertension (*e.g.*, collateral development) were not observed. Right lobectomy accompanied by regional lymphadenectomy was proposed, and the estimated liver remnant against the whole liver was 47.1%. Operative time was 238 min; blood loss was 531 mL; blood transfusion was not required; and curative surgery for ICC was successfully achieved. The patient's postoperative course was uneventful, and she was discharged 13 days after surgery.

**Fig. 2:**
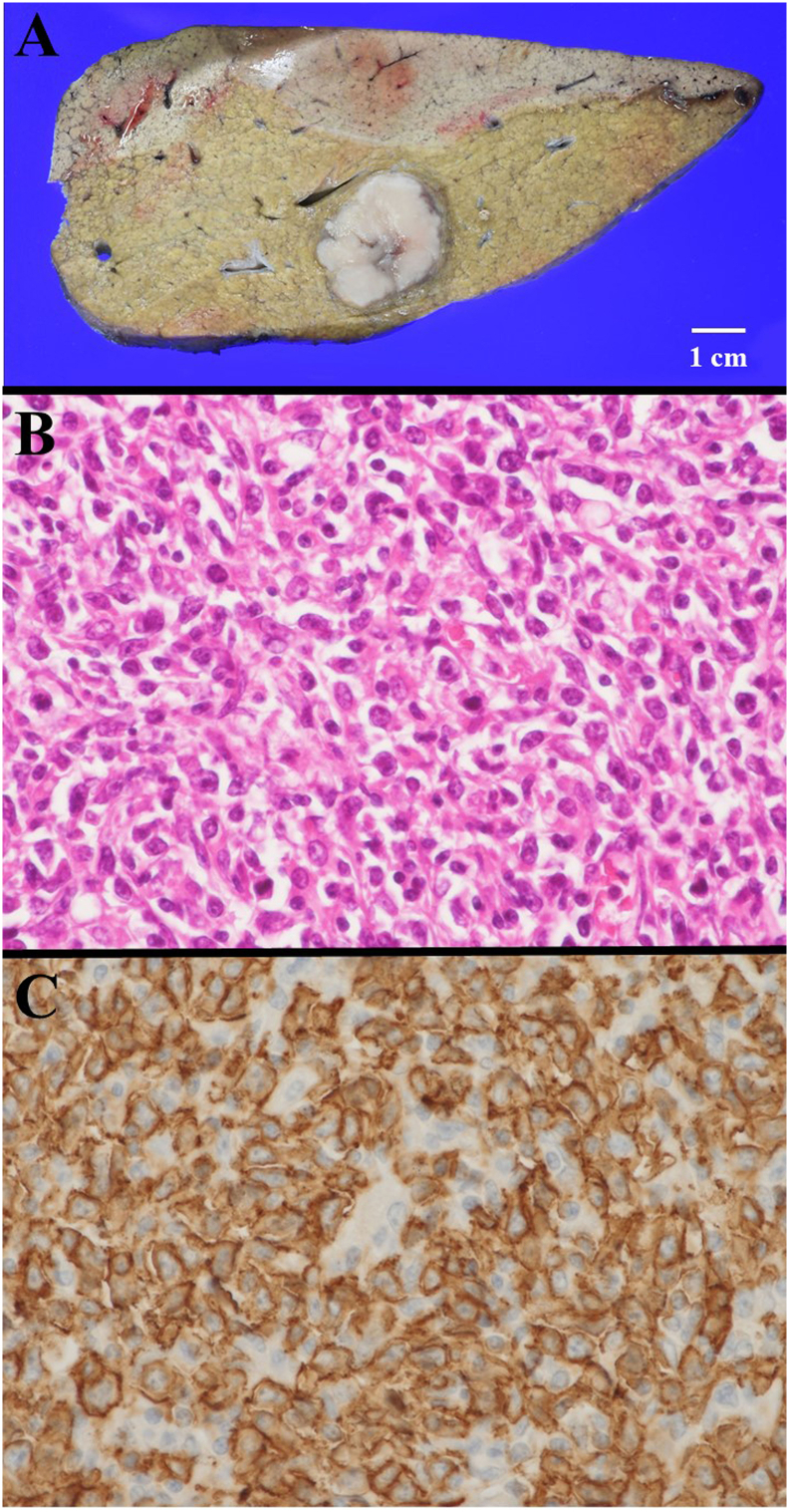
Pathological assessment Macroscopic findings are shown (A). Pathological findings with hematoxylin and eosin staining (×400) are shown (B). In the immunohistochemical examination, tumor cells were positive for CD20 (C). Unexpectedly, a definitive diagnosis of DLBCL was made pathologically. Abbreviation: DLBCL, diffuse large B-cell lymphoma.

Unexpectedly, the pathological assessment definitively diagnosed DLBCL (Fig. 2) not invading the vessels and bile duct, and the macrovascular thrombosis in the PV was not tumor thrombosis but a blood thrombus. With immunohistochemistry, the DLBCL was positive only for CD20 and negative for hepatocyte-specific antigen, CD3, CD31, and CD34. Lymphoid metastasis was not observed in the harvested LNs. The liver parenchyma was pathologically assessed as cirrhosis (A2F4) according to a scoring system [6]. Although the serum lactic dehydrogenase concentration was normal, soluble interleukin-2 receptor concentration was increased at 845 U/mL. Deoxyribonucleic acid evaluation for Epstein–Barr virus was normal in the quantitative determination using a blood sample.

**Fig. 3:**
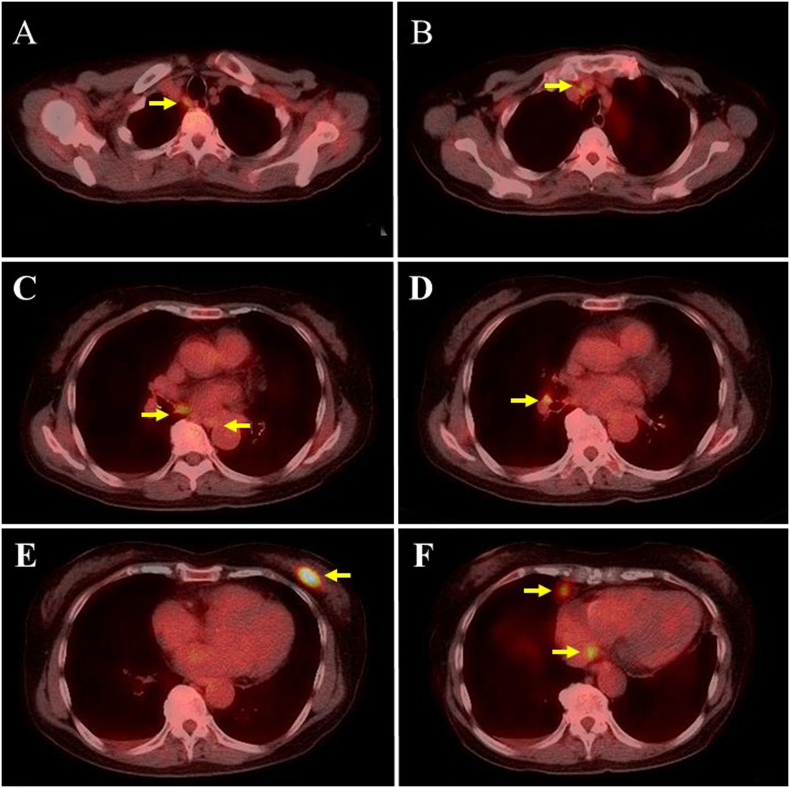
Abnormal accumulation during positron emission tomography-computed tomography Positron emission tomography-computed tomography images showing abnormal accumulations (yellow arrows) in the peritracheal lymph nodes (LNs) (A, C, and D), parasternal LNs (B and F), mediastinal LNs (C and F), breast tissue (E), and pericardial LNs (F) even though these LNs were small and not enlarged. (For interpretation of the references to colour in this figure legend, the reader is referred to the web version of this article.)

**Fig. 4:**
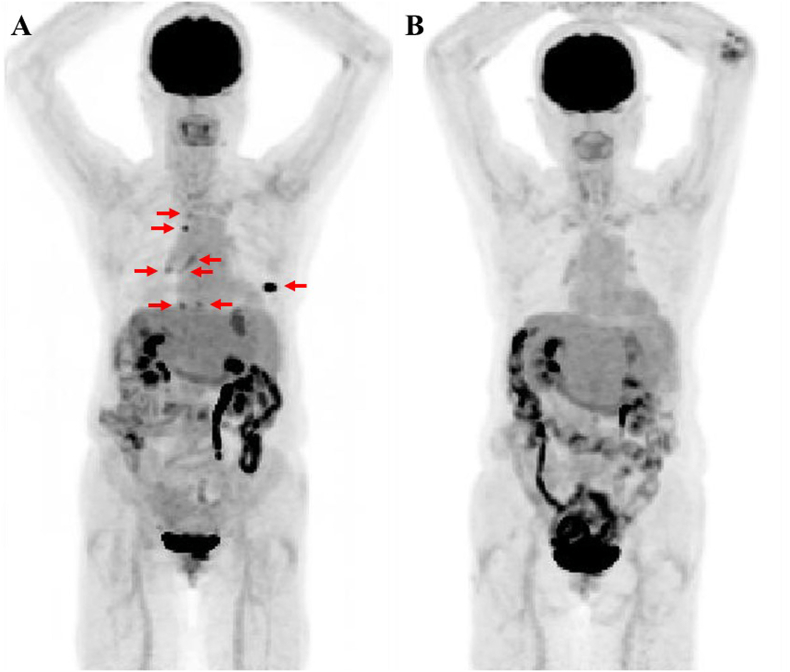
Fluorine-18-fluorodeoxyglucose positron emission tomography findings before and after systemic chemotherapy Abnormal accumulations are seen in the images obtained before systemic chemotherapy (red arrows), which all clearly disappeared after systemic treatment. (For interpretation of the references to colour in this figure legend, the reader is referred to the web version of this article.)

Fluorine-18-fluorodeoxyglucose positron emission tomography-computed tomography was performed after obtaining the definitive diagnosis. Abnormal accumulations were clearly observed in the breast tissue and peritracheal, parasternal, mediastinal, and pericardial LNs (Fig. 3, Fig. 4A) even though these were small LNs that were not enlarged. The patient received six cycles of rituximab, pirarubicin, cyclophosphamide, vincristine, and prednisolone (R-THP-COP regimen) from 49 days after surgery, and achieved complete remission (Fig. 4B). She is being closely followed bimonthly, and has had no recurrence for 3 years after surgery.

## 3 Discussion

The two most frequent primary liver neoplasms are HCC and ICC [1], and PV tumor thrombosis is more common with HCC than with ICC [7,8]. Tumor thrombosis into the PV is a clear factor indicating a poor prognosis, even after extended hepatectomy and liver transplantation [[7], [8], [9]]. In our case, the characteristic sign of a dynamic enhancement pattern (early wash-in and wash-out) in HCC was not observed; therefore, we made a preoperative diagnosis of ICC with PV tumor thrombosis.

Primary liver lymphoma is rare and accounts for <0.1% of malignant liver tumors [10]. In our case, we could not confirm the tumor as primary liver lymphoma because extrahepatic lesions were observed. It is known that DLBCL generally does not invade vessels [2], and our case showed no tumor thrombosis in the PV, although macrovascular thrombosis appeared to be tumor thrombosis in preoperative imaging.

Autoimmune disorders may increase the risk of lymphoma [2], especially non-Hodgkin's lymphoma [2,3]. Regarding the development of lymphoma in PBC patients, non-Hodgkin's lymphoma constitutes approximately 70% of these lymphomas. However, the association between DLBCL and PBC is still unclear [2], and the rate of lymphoma in patients with PBC is estimated at only 0.6% [2]. Regarding non-Hodgkin's lymphoma in PBC patients, there are only a few case reports of DLBCL [2,3], mucosa-associated lymphoid tissue lymphoma [11,12], and cutaneous T-cell lymphoma [13].

PBC is an autoimmune disease characterized by destruction of intrahepatic bile ducts which results in progressive damage to the biliary tree, cholestasis and ultimately advanced liver disease [14]. Patients with autoimmune disorders seem to have an elevated risk of lymphoma, especially non-Hodgkin's lymphoma [2,3,[11], [12], [13]]. For PBC patients, advances in practice have currently improved clinical care, driven novel therapeutic options and developed risk stratification tools [14], though a number of immunosuppressants may not be associated with clinical benefit [2,3]. The use of more potent immunomodulatory agents may raise concerns over the risk of adverse effects including the development of non-Hodgkin's lymphoma [2,3,[11], [12], [13]]. The increased risk has been attributed to the disturbance of immunological system found in these patients or to the immunosuppressive therapy used to treat the autoimmune disorders [2,3,[11], [12], [13]].

In present case, intraoperative pathological examination could be chosen to avoid invasive surgery. Liver needle biopsy could be performed percutaneously or intraoperatively, even though there is a concern for potential dissemination and bleeding along with tumor biopsy. In fact, we did not consider the possibility of DLBCL preoperatively in our patient, and her definitive diagnosis was made pathologically. Even though postoperative complication fortunately didn't occur in our patient, it is a regrettable mistake in our case that we didn't have any considerations for liver needle biopsy before or during surgery because of preoperative assumption of ICC. We must remember that DLBCL may develop rarely in PBC patients. We believe our detailed case presentation provides a timely reminder for clinicians and surgeons in the fields of hepatology and hematology.

## 4 Conclusions

We presented a thought-provoking case of a difficult-to-diagnose liver tumor with PV thrombosis in a PBC patient.

## Actual documents for IRB approval and publication consent

Both actual proof of IRB approval and signed consent form for publication are also submitted.

## Provenance and peer review

Not commissioned, externally peer-reviewed.

## Sources of funding

None. This research did not receive any specific grant from funding agencies in the public, commercial, or not-for-profit sectors.

## Ethical approval

Data were retrospectively evaluated. This report was approved by the Institutional Review Board of Shiga General Hospital, Moriyama, Japan.

## Consent

Written informed consent was obtained from the patient for publication of this case report and accompanying images. A copy of the written consent is available for review by the Editor-in-Chief of this journal on request.

## Research registration (for case reports detailing a new surgical technique or new equipment/technology)

None.

## Guarantor

None.

## CRediT authorship contribution statement

Tomohide Hori, PhD., MD., FACS. wrote the manuscript. H. Katsura and T. Hori collected the data. All authors analyzed the data, discussed therapeutic options, reviewed previous papers, and provided important opinions. T. Hori and M. Zaima supervised this report. H. Katsura and T. Hori contributed equally to this work.

## Declaration of competing interest

None of the authors have any financial conflicts of interest to declare.
